# Ovarian Cancer Classification based on Mass Spectrometry Analysis of Sera

**Published:** 2007-02-17

**Authors:** Baolin Wu, Tom Abbott, David Fishman, Walter McMurray, Gil Mor, Kathryn Stone, David Ward, Kenneth Williams, Hongyu Zhao

**Affiliations:** 1 Department of Epidemiology and Public Health; 2 W.M. Keck Biotechnology Resource Laboratory; 3 Department of Obstetrics and Gynecology; 4 Department of Genetics, Yale University School of Medicine, New Haven, CT, USA; 5 Department of OB/GYN, New York University School of Medicine, New York, NY, USA; 6 Division of Biostatistics, School of Public Health, University of Minnesota, Minneapolis, MN, USA

## Abstract

In our previous study [1], we have compared the performance of a number of widely used discrimination methods for classifying ovarian cancer using Matrix Assisted Laser Desorption Ionization (MALDI) mass spectrometry data on serum samples obtained from Reflectron mode. Our results demonstrate good performance with a random forest classifier. In this follow-up study, to improve the molecular classification power of the MALDI platform for ovarian cancer disease, we expanded the mass range of the MS data by adding data acquired in Linear mode and evaluated the resultant decrease in classification error. A general statistical framework is proposed to obtain unbiased classification error estimates and to analyze the effects of sample size and number of selected m/z features on classification errors. We also emphasize the importance of combining biological knowledge and statistical analysis to obtain both biologically and statistically sound results.

Our study shows improvement in classification accuracy upon expanding the mass range of the analysis. In order to obtain the best classification accuracies possible, we found that a relatively large training sample size is needed to obviate the sample variations. For the ovarian MS dataset that is the focus of the current study, our results show that approximately 20–40 m/z features are needed to achieve the best classification accuracy from MALDI-MS analysis of sera. Supplementary information can be found at http://bioinformatics.med.yale.edu/proteomics/BioSupp2.html.

## Introduction

Proteomics is an integral part of the process of understanding biological systems, pursuing drug discovery, and uncovering disease mechanisms. Because of their importance and their very high level of variability and complexity, the analysis of protein expression and protein:protein interactions is as potentially exciting as it is a challenging task in life science research [2]. Comparative profiling of protein extracts from normal versus experimental cells and tissues enables us to potentially discover novel proteins that play important roles in disease pathology, response to stimuli, and developmental regulation. However, to conduct massively parallel analysis of thousands of proteins, over a large number of samples, in a reproducible manner so that logical decisions can be made based on qualitative and quantitative differences in protein content, is an extremely challenging endeavor.

Mass Spectrometry (MS) is being used increasingly for rapid identification and characterization of protein populations. Recently, there has been extensive research directed toward the utilization of MS technology to build molecular diagnosis and prognosis tools for cancers [3,4,5]. Many of the papers have claimed ≥90% sensitivity and specificity using a subset of selected m/z features; some of them even achieve perfect classification [6]. But upon close inspection of some of these studies, some of the identified m/z features correspond to background noise, which suggests some systematic bias from non-biological variation in the dataset [12,13,14]. In our opinion many of these studies do not give sufficient importance to data pre-processing and to the appropriate interpretation of the MS data. Another commonly neglected area is the correct way of using cross-validation (CV). As discussed in [7], it is important to carry out an external CV, whereby at each stage of the validation process *no* information is used from the testing set to build a classifier from the training set. Internal CV is used in many current MS studies, whereby the selection of m/z features has utilized information from all the samples, which will *substantially* under-estimate classification error. In our previous study [1], our goal was to compare the relative performance of popular classification methods in the context of an MS ovarian cancer dataset. For ease of comparison we chose a subset of fixed features before we compared classification methods. This internal CV will most likely prove to seriously under-estimate classification errors. For the current ovarian cancer data, we have found that (see data on the supplementary website) the *relative* performance ranking of the different methods tested previously [1] was not changed by using external versus internal CV. These results again support the good performance of the random forest (RF) [8] approach when compared to other classification methods. In this study we use RF to estimate the unbiased classification error for our ovarian cancer MS data which is derived from MALDI-MS analysis of desalted sera samples. In the meantime, we also empirically evaluate the impact of the number of selected m/z features and the sample size on classification error. Our analysis framework provides a general guideline for the practice of utilizing MS for cancer or other disease molecular diagnosis and prognosis.

## Experimental Data

We have obtained ovarian cancer and control serum samples from the National Ovarian Cancer Early Detection Program at Northwestern University Hospital. The Keck Laboratory then subjected these samples to automated desalting and MALDI-MS on a Micromass M@LDI-L/R instrument as described generally at: http://info.med.yale.edu/wmkeck/prochem/biomarker.htm.

The M@LDI-L/R mass spectrometer automatically acquires two sets of data in positive ion detection mode. The mass range acquired is dependent on the mass analyzer being used, with 700–3500 Da for reflectron and 3450–28000 Da for linear. The resulting merged dataset consists of MS spectra that extend from 700 to 28000 Da and were obtained on serum samples from 93 ovarian cancer patients and 77 normal samples.

## Methods

### Classification Error Estimation with RF

RF [8] combines two useful features: Bootstrap to produce pseudo-replicates and random feature selection to improve prediction accuracy. Each bootstrap sample (sampled with replacement from the original sample) is used to generate a classification tree; and the final classifier is based on voting from all the trees obtained from all the bootstrap samples. Generally the larger the number of trees, the more accurate the error rate estimation. In the construction of classification trees, at each node split (i.e., branch) the best m/z feature is selected from a random subset of all the features. After the first split the two resulting daughter populations of samples are “purer” in the sense that they have higher or lower fractions of cancer samples. Each of the two resulting branches is then split into another two branches at another m/z feature whose relative level of intensity can be used to best separate cancer from control samples. Additional nodes (i.e., with each occurring at an m/z value that has the best ability to accurately classify the samples in that particular daughter population) will be found and utilized until each of the resulting daughter populations is 100% “pure” such that each contains only control or cancer samples. The random selection of features is an innovation introduced in RF as a way to reduce the dependence between classification trees, which in turn may lead to the reduction of the classification errors [8]. Typically, a “forest” might have 5,000 trees and each tree might require >20 branches depending on the sample size. RF can also estimate the importance of features based on their individual contribution to the classifications. For the detailed algorithm, see [1]. We use the R [15] interface of random forest [16] in our analysis. From the RF program we can derive the posterior probability of each sample belonging to each class. Based on these posterior probabilities we can evaluate the sensitivity, specificity and classification errors based on RF.

We can summarize our MS dataset for *n* samples in a *p* by *n+*1 matrix: (*mz*, ***X)*** = (*mz*, *X*_1_, …, *X_n_*) where *p* is the number of m/z ratios observed, *mz* is a column vector denoting the measured m/z ratios, and the *x*_i_ are the corresponding intensities for the i-th sample. We use vector ***Y*** = (y_i_) to denote the sample cancer status. Our goal is to predict y*_i_* based on the intensity profile *X’*_i_ = (*x*_1_*_i_*, *x*_2_*_i_*, …, *x_pi_*). Assume that we have *g* classes. RF classifier partitions the space ***X*** of protein intensity profiles into *g* disjoint subsets, A_1_, …, A_g_, such that for a sample with intensity profile X = (x_1_, …, x*_p_*) ∈ A_j_ the predicted class is j.

Classifiers are built from observations with known classes, which comprise the *learning set* (*LS*) *L* = {(X_1_, y_1_), …, (Xn_L_, yn_L_)}. Classifiers can then be applied to a *test set* (*TS*) *T* = {X_1_, …, Xn_T_}, to predict the class for each observation. If the true classes *y* are known, they can be compared with the predicted classes to estimate the error rate of the classifiers.

We denote the RF classifier built from a learning set *L* by ***C***(., *L*). Given a new sample (X, y), we can represent ***C***(X, *L*) by a *g*-element vector (C_1_, …, C_g_). If we want a hard-decision classifier, we will have C_k_ = 1 and C_i≠k_ = 0, i.e. it predicts sample (X, y) to belong to class k. Or we also can obtain a probability output, Pr(C_i_ = 1) = P_i_∈[0,1] and ∑_i=1, …, g_ P_i_ = 1, i.e. it predicts the probability that sample (X, y) belongs to class k is P_k_.

For our ovarian cancer dataset we only have two classes, cancer (y = 1) and normal (y = 2) samples. For two-class classification problems we can define sensitivity (θ) and specificity (η), which are inherently related to classification errors. Sensitivity is also known as the true positive rate, which is the probability of classifying a sample as cancer when it actually derives from a patient with cancer, i.e. Pr(***C***(X, *L*) = 1|y = 1). Specificity is also known as the true negative rate, which is the probability of classifying a sample as normal when it is actually from a normal sample, i.e. Pr(***C***(X, *L*) = 2|y = 2). The relationship between sensitivity and 1-specificity is well known as a ROC curve in medical research.

If ***C***(X, *L*) is a hard-decision classifier, we can estimate sensitivity and specificity using sample proportions,

$$\begin{array}{l} \hat{\theta }=\frac{\sum_{i=1}^{n}I\{y_{i}=1\}I\{C(X_{i},L)=1\}}{\sum_{i=1}^{n}I\{y_{i}=1\}},\\ \hat{\eta }=\frac{\sum_{i=1}^{n}I\{y_{i}=2\}I\{C(X_{i},L)=2\}}{\sum_{i=1}^{n}I\{y_{i}=2\}}. \end{array}$$

The most commonly used classification error (Err) is estimated as

$$\begin{array}{l} \hat{Err}=\frac{\sum_{i=1}^{n}I\{C(X_{i},L)\neq y_{i}\}}{n}\\ =\frac{n_{1}}{n}\sum_{i=1}^{n}I\{C(X_{i},L)=2,y_{i}=1\}\\ +\frac{n_{2}}{n}\sum_{i=1}^{n}I\{C(X_{i},L)=1,y_{i}=2\}\\ =\frac{n_{1}}{n}(1-\hat{\theta })+\frac{n_{2}}{n}(1-\hat{\eta }), \end{array}$$

where n_1_ and n_2_ are the sample size for the cancer and normal groups, 1-θ is classification error for the cancer group, and 1-η is the classification error for the normal group. If we have a very un-balanced sample set, i.e. n_1_>> n_2_ or n_1_>> n_2_, we can see that the previous definition of Err will encourage classifying all samples into the group with the larger sample size, which obviously is not the optimum approach. To avoid this problem we can use a balanced classification error definition

$$\begin{array}{l} \hat{Err}=\frac{1}{2}(1-\hat{\theta })+\frac{1}{2}(1-\hat{\eta })\\ =\frac{1}{2}\sum_{i=1}^{n}I\{C(X_{i},L)=2,y_{i}=1\}\\ +\frac{1}{2}\sum_{i=1}^{}I\{C(X_{i},L)=1,y_{i}=2\}. \end{array}$$

This error definition gives equal weights to the cancer versus control groups and makes the subjective assumption that specificity and sensitivity are equally important. If the latter assumption is not optimal for a particular application, the above equation allows for easily changing the relative importance of specificity versus sensitivity depending upon the experimental context.

In the case where we have a probability output, we first select a threshold α and then define the hard-decision classifier as

$$C(X_{i},L)=\{\begin{array}{l} 1\;\text{if }P_{1,i}\geq \alpha \\ 2\;\text{otherwise} \end{array}.$$

We can then estimate θ, η and Err similarly as before

$$\begin{array}{l} \hat{\theta (\alpha )}=\frac{\sum_{i=1}^{n}I\{y_{i}=1\}I\{P_{1,i}\geq \alpha \}}{\sum_{i=1}^{n}I\{y_{i}=1\}},\\ \hat{\eta (\alpha )}=\frac{\sum_{i=1}^{n}I\{y_{i}=2\}I\{P_{1,i}\geq \alpha \}}{\sum_{i=1}^{n}I\{y_{i}=1\}}\text{and}\\ \hat{Err(\alpha )}=\frac{1}{2}\left(1-\hat{\theta (\alpha )}\right)+\frac{1}{2}\left(1-\hat{\eta (\alpha )}\right). \end{array}$$

As mentioned above, the relationship between $\hat{\theta (\alpha )}$ and $\hat{\eta (\alpha )}$ is the commonly used ROC curve. Minimum classification error can be estimated as min_α ∈ [0,1]_ $\hat{\text{Err}(\alpha )}$. Our choice of balanced error rate as the representation is just one way to deal with an unbalanced sample size. The full information is included in the ROC curve, which enables us to choose different specificity and sensitivity depending on the context of specific problems.

### Pre-processing

The purpose of pre-processing, arguably the most important step in MS data analysis, is to reduce the effects of noise and to facilitate interpretation of the MS datasets. Before we submit the dataset to our final classifier, we carry out the following preprocessing steps: mass alignment, normalization, smoothing and identification of m/z data points that occur in peaks. The detailed pre-processing steps used in the current work are discussed in [17]. More advanced methodologies for peak alignment are discussed in [9,10].

Briefly, mass alignment is accomplished by numbering reflectron data points consecutively (in both a positive and negative direction) by assigning the observed m/z value that is closest to the expected MH^+^ for the C12 isotope of the internal standard Bradykinin, which is 1060.569, as data point zero. A similar approach is then used with the linear data points which are aligned by assigning the m/z data point closest to the expected average MH^+^ of oxidized insulin chain B, which is 3496.95, as data point 100,000. Spectra are normalized (so they each contribute more equally to the overall search for biomarkers), by determining a linear normalization factor for each spectrum which minimizes the summed difference between all intensities observed within the specified reflectron + linear mass range and the corresponding intensities in the *overall*, baseline-corrected median spectrum for all samples. Baseline smoothing begins by taking the natural log of all intensities. A sliding window of 1,000 data points is then used to determine the least squares, robust local polynomial fit curve that best represents the baseline. We then subtract the corresponding baseline intensity for the polynomial fit curve from each data point. Finally, we assume that only data points in completely or partially resolved peaks result from peptide or small protein ions and are likely to be meaningful. To be classified as occurring in a peak, at least 3 of 4 successive data point intensities before or after each candidate marker data point must show a progressive increase or decrease in background corrected, normalized peak intensity. Each peak data point is then extended by an additional 4 data points before/after the last data point that passed the peak test. Hence at this point we include a band width around each peak.

### CV

Since we do not have a test set, CV was utilized to provide a nearly unbiased estimate of the classification error. The idea of CV is to randomly partition the original data into two parts: a training set used to build the classifier, and a testing set used to estimate the performance of the classifier. As discussed in [7], the commonly used leave-one-out CV approach has high variance. K-fold CV is recommended, whereby K is usually taken to be around 5 or 10. In our study we use 5-fold CV to estimate classification errors. It is important to perform peak identification and biomarker selection inside each CV to avoid selection bias and to obtain an unbiased classification error estimation.

### Study Design

It is obvious that Err depends on the underlying classifier, sample size *N* and the number of selected m/z features *M*. In this study we have fixed the classifier to be RF. We will evaluate the impact of *N* and *M* on Err. Our strategy is to model empirically the functional relationship Err(*N*, *M*) for a grid of values of *N*, *M*. For MS data the total number of features is usually very large, there are a total p = 130,000 m/z ratios for each of our ovarian cancer Linear + Reflectron spectra and the total number of selected m/z features is usually in the range of 10 ~ 100. In our study we evaluate Err for *M* ranging from 5 to 100. The total number of samples is usually very small compared to the total number of features. There are a total of n = 170 samples in our current ovarian cancer dataset. We need to carry out an extrapolation to estimate the impacts of *N* on Err. As discussed in [10], there is an inverse-power-law learning curve relationship between Err and *N*, Err(N) = *β* _0_ + *β* _1_*N*^−α^, which is approximately true for large sample size data-sets (usually about tens of thousands of samples). β_0_ is the asymptotic classification error and (α, β_1_) are positive constants. Our current dataset has a relatively small sample size (n = 170) compared to its high-dimension feature space (p = 130,000). Under this situation it is very dangerous to rely on the learning curve model to extrapolate β_0_, which corresponds to an infinite training sample size *N* = ∞. But within a limited range we can still rely on this model to extrapolate the classification error to full sample size n = 170. To estimate parameters (α, β_0_, β_1_), we need to obtain at least three observations. In our use of 5-fold CV to estimate classification errors, we first used one of the groups as a testing set, which produces a training set of *N* = 170/5*4 = 136 samples. We then use two, three and four of the groups as testing sets, which will give *N* = 102, 68, 34. For each *N* we estimate classification errors with *M* = 5, 6, …, 100 m/z features. We can estimate the learning curve based on these errors.

## Results

### Classification Error Estimation

**Figure 1:**
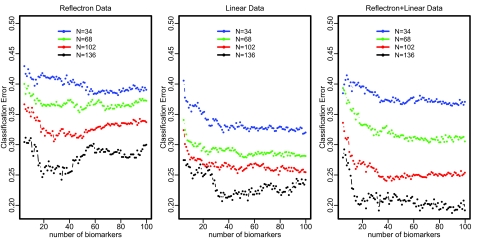
Five-fold CV Estimation of Err(N, M) for Ovarian Cancer MALDI-MS Data. N is the sample size of the training set, and M is the number of m/z features selected.

Figure 1 displays the 5-fold CV classification error estimations for our ovarian cancer data. The optimal classification error is about 25% for Reflectron data, and 21% for Linear data. Combining Reflectron and Linear data, the optimal classification error is reduced to about 19%. For relatively smaller sample size (N = 34, 68), the classification errors for Linear data increase after adding the Reflectron data, which reflects the variation due to smaller sample size and the impact of noise from Reflectron data. For larger sample size (N = 102,136), the combination of Reflectron and Linear data outperforms the Linear data. These classification error comparisons reflect that the contribution for sample classification mainly comes from the Linear data. Overall we can see clearly the trend that a larger training set has smaller classification errors. And for a fixed training set, classification error drops dramatically from 5 to 20 m/z features and then it levels off at about 20–40 m/z features for the combined Reflectron+Linear data. With 136 samples in the training set, we can achieve about 19% classification error. We use a learning curve to extrapolate Err(170, *M*) for each *M*. Please see the supplementary website for numerical values of the learning curve parameter estimations.

**Figure 2:**
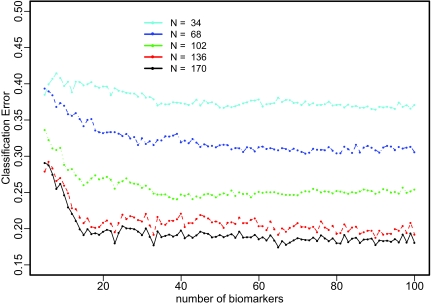
Classification Error Extrapolation for Reflectron+Linear MALDI-MS Data. N is the sample size of the training set; and M is the number of m/z features selected.

Figure 2 displays the estimated classification errors for total sample size n=170. We can see that there are large improvements when the sample size is increased from 34 to 68 and then to 102. But there is not much further improvement predicted to occur on going from 136 samples to 170 samples. Overall, the classification error levels off after 20 to 40 m/z features at an optimal classification error of about 18%.

### Identification of m/z Data Points Whose Intensities Contribute Towards the Correct Classification of Cancer versus Control Sera

**Figure 3–6:**
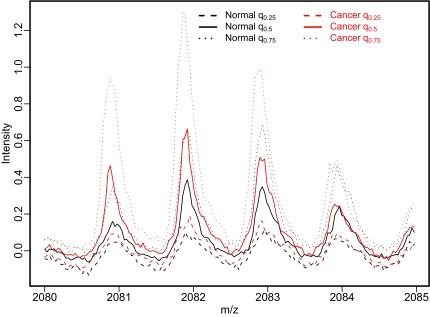

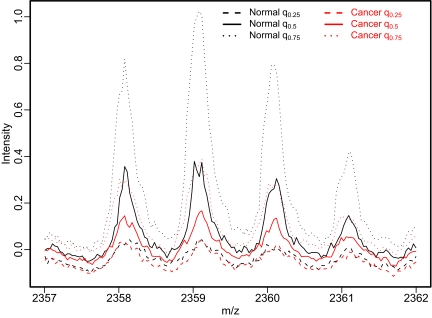

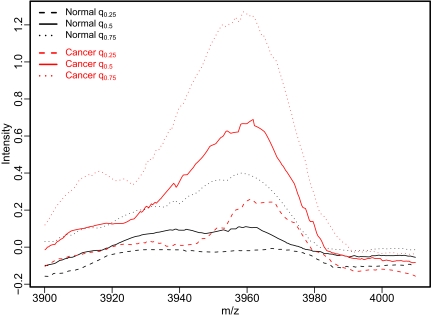

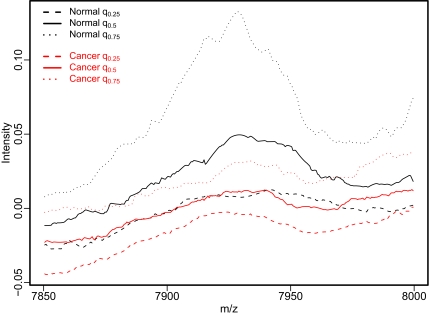
Local Exploration of Identified m/z features. q0.25 is the first quartile of the intensities, q0.5 is the median, and q0.75 is the third quartile.

One of the major interests in utilizing MS data is to identify important m/z features to build molecular diagnosis and prognosis tools. As discussed in [1], the RF program has some advantages over traditional T-statistics for biomarker identification in terms of minimizing classification errors. Here we apply RF to our 170 ovarian cancer samples to rank important m/z features. To guard against false positives, it is very important to explore the local behavior of the identified m/z features. To explore the relative intensities of all samples in one figure will make the plot obscure. Instead, we visually compare median, first and third quartile intensities of normal and cancer groups in one plot. In the following biomarker exploration plots (Fig. 3–6), q_0.25_ is the first quartile intensity, q_0.5_ the median intensity and q_0.75_ the third quartile intensity. We can clearly see the difference between cancer and normal groups. However, there is no single m/z feature that can completely distinguish cancer from normal groups; rather, there are considerable overlaps between these two groups. For some m/z features the normal group has higher intensities, while the cancer group dominates at other m/z features.

For the 40 identified m/z features, some of them originate from the same peptide ion. In the following we show four unique and representative m/z features, which (especially in the case of the reflectron data in Fig. 3 and 4) visually appear to result from the ionization of biologically meaningful peptides. In Figure 3–6, regions around 4 m/z features are plotted, where the green lines indicate the exact m/z position of each potential biomarker as determined by the RF algorithm.

## Discussion

In this paper we provide an unbiased estimate of the classification error rate for an ovarian cancer dataset composed of a single reflectron or merged reflectron + linear MALDI-MS spectrum for each serum sample. With Reflectron data alone, we can achieve about 25% classification error. After expanding the mass range of the MS data with the use of the Linear analyzer, the optimal classification error achieved with 170 samples decreased to about 18%.

Our goal in adding the linear data was to reduce the classification error by expanding the mass range – which enabled the detection of higher mass biomarkers (i.e., see Figures 5–6) that would not have been detected with a reflectron analyzer. Rather than using only linear spectra, which also could have covered the entire 700 Da to 28,000 Da range, we decided to use reflectron data for the low mass region. Our reasoning for taking this approach is that if there are overlapping peptide spectra, the use of high resolution reflectron data might better enable an m/z datapoint to be found (within an individual peptide ion envelope) that is not partially compromised by resulting from ionization of both the biomarker of interest as well as an unrelated peptide ion. In this regard, it is interesting to note that the optimal m/z for the biomarker whose spectrum is shown in Fig. 4 is not at the position of the monoisotopic ion, which would be close to 2358. Rather, the optimal m/z for this peptide biomarker is close to 2361. Another very important message is the correct use of CV for estimating prediction errors. While some other cancer studies using MS data have reported nearly perfect classifications, they usually result from internal CV which will produce serious underestimates of the actual classification error, e.g. in our previous study [1], the optimal internal classification error is about 8% compared to the “external” classification error of 25%.

From our analysis, the number of m/z features has a large impact on the prediction accuracy. The optimal number of m/z features certainly depends on the properties of different datasets. For our current ovarian cancer MS data, 20–40 m/z features seem to achieve a balance between noise and signal. Our proposed CV methods provide a general framework for diagnosing the impact of the number of m/z features on the classification error.

Another neglected aspect in many current studies is the justifications for numerically identified m/z features as being biologically meaningful. We agree that when classification errors begin to be sufficiently low to allow the corresponding m/z features to be clinically useful, then it would be advisable to identify the protein origin of those markers. Since we did not find any significant m/z feature that was specific to either the cancer or control sample spectra, we imagine that many of these markers may derive from proteins that are not involved in the disease process being studied and that they may prove to result from secondary effects (e.g., the disease being studied may produce higher than normal levels of proteases which might be released into the blood stream and cause unusually high extents of cleavage of some serum proteins). While these types of m/z features may have little or no value in furthering our understanding of the disease process itself, this would not lessen the value of these m/z features for properly classifying unknown samples. It also is important to point out that we do not currently know if the m/z features whose relative intensities enabled a >80% success rate at classifying ovarian cancer versus control sera samples are indeed specific to *ovarian* cancer. Finally, it is important also to note that our 80% success rate is applicable only to the analyzed sample set which, because it was composed of approximately equal numbers of sera from ovarian cancer versus control samples, is not representative of the general population. Nonetheless, we are extremely encouraged by the results of these preliminary studies and currently believe that the best way to improve the dynamic range of the MALDI-MS analyses (and the predictive ability of the resulting biomarkers”) upon which our classifications are based is by pre-fractionation of sera samples and the subsequent analysis of multiple spectra/sample.
